# Pediatric laryngeal schwannoma: a case report

**DOI:** 10.1093/jscr/rjad587

**Published:** 2023-10-21

**Authors:** Yaser A Hamam, Basel F Alqeeq, Mohammed Hamam, Ahmed M Abuhelal, Saleh A Saqer

**Affiliations:** Faculty of Medicine, Al-Quds University, Jerusalem, Palestine; Faculty of Medicine, Islamic University of Gaza, Gaza, Palestine; Faculty of Medicine, Islamic University of Gaza, Gaza, Palestine; Otolaryngology Department, Nasser Medical Complex, Gaza, Palestine; Otolaryngology Department, Nasser Medical Complex, Gaza, Palestine

**Keywords:** schwannoma, neurilemmoma, laryngeal, laryngoscopy, neurogenic tumor

## Abstract

Schwannomas are benign tumors originating from Schwann cells in the peripheral nervous system. They mostly occur in the head and neck region but are rare in the larynx, and present with various symptoms. Surgical removal is the recommended treatment. This study presents a 12-year-old female with sudden onset hemoptysis, snoring, difficulty breathing, dysphagia, and voice changes. On examination, she was conscious and had muffled voice while speaking, without signs of respiratory distress. Endoscopic laryngoscopy revealed a large laryngeal mass obstructing the vocal cords. Endoscopic excision and biopsy confirmed the presence of the laryngeal schwannoma. Postoperative recovery was uneventful, with normal vocal cord function and no recurrence at follow-up.

## Introduction

Schwannoma is a benign, encapsulating, neurogenic tumor, originating from the peripheral nervous system's Schwann cells. Approximately 25–45% of all schwannomas occur in the head and neck region. Parapharyngeal spaces represent the most common source [1]. Nevertheless, the literature reports few cases of schwannoma tumors of the larynx. As it accounts for 0.1% of all benign laryngeal neoplasms [2]. Schwannomas can affect individuals of all age groups, but they are most commonly identified in patients between the ages of 20 and 50, with a variable distribution among the genders [3–5]. Schwannomas are not commonly related with specific lifestyles or habits. Genetic predisposition and prior exposure to radiation are documented risk factors for schwannoma [5]. Laryngeal Schwannomas may present with various clinical pictures such as dysphagia, dysphonia, odynophagia, hoarseness, the presence of a lateral neck lump, globus sensation (a feeling of a lump in the throat), and dyspnea [6–8]. The recommended investigation, high-definition video laryngoscopy, typically identifies a submucosal tumor that sometimes obscure the true vocal cords, impairing their mobility. Of note, histopathology is still the gold standard in diagnosis. As laryngeal schwannomas are not sensitive to radiotherapy or chemotherapy, the only effective treatment is surgical resection [9]. The aim of this report is to present and document the clinical presentation, diagnostic approach, and management for such a rare tumor.

## Case presentation

A 12-year-old female patient attended the emergency room of Nasser Medical Complex on 11/8/2022 complaining of hemoptysis 4 days prior to presentation. The patient reported multiple episodes of hemoptysis which were sudden in onset and associated with snoring, difficulty of breathing, dysphagia, and generalized fatigue. Over the past 6 months, she had intermittent attacks of dry cough. Furthermore, her family noticed a change in her voice recently. There were no other associated systemic symptoms. Her past-medical and surgical history are unremarkable.

**Figure 1:**
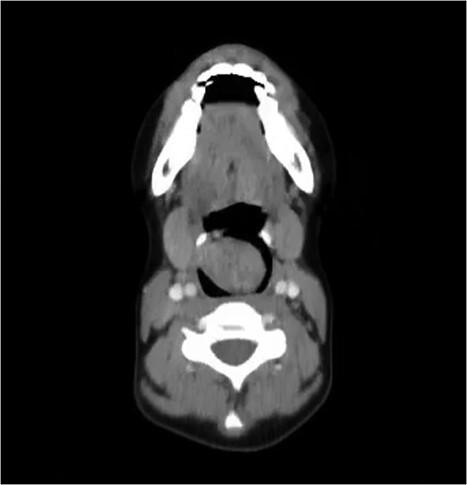
CT of the neck with axial cut showing the schwannoma occupying the larynx.

On examination, she was conscious, vitally stable and without any signs of respiratory distress. She was found to have a muffled voice while speaking. Upon inspection, using a tongue depressor, a part of a mass could be slightly visualized with gag reflex. Therefore, a consultation with the otolaryngology department was made for further evaluation. The patient underwent endoscopic laryngoscopy which revealed a large whitish mass that occupies the laryngeal inlet, blocking the view of the vocal cords. Computer tomography (CT) showed a well circumscribed (4 × 3 cm), low-attenuation, midline supraglottic mass, commencing just below the aryepiglottic folds and extending onto sclerotic arytenoids (Fig. 1).

Written informed consent was obtained from the patient. Under general anesthesia, bougie-assisted tracheal intubation via video laryngoscopy was performed. Endoscopic excision of the whole mass was performed with preservation of the adjacent mucosa. Then, a biopsy was sent for histopathological evaluation. The patient was extubated after the procedure, without a need for tracheostomy and was subsequently discharged safely 3 days later.

Histopathological examination revealed typical pathological characteristics of schwannoma. First, compact hypercellular Antoni A areas along with myxoid hypocellular Antoni B areas were identified by light microscopy. Second, a positive reaction to S-100 stain was evident by strong and diffuse staining.

Postoperative laryngoscopy revealed normal vocal cord mobility. At 3- and 6-month follow-up visits, neither a recurrence nor a residual mass was found in this patient. She was able to swallow and breathe normally once again, and her voice has returned to normal.

## Discussion

In this study, we reported a rare case of a schwannoma tumor occupying the larynx and presenting with obstructive symptoms and hemoptysis.

Laryngeal Schwannoma or neurilemmoma is a rare benign encapsulated neurogenic tumor. The first diagnosis of laryngeal schwannoma goes back to 1925 by Schwanck [10]. Laryngeal schwannomas typically originate in supraglottic space; either from the true or false vocal cords or the aryepiglottic fold [11]. However, rare cases have been found to arise in the subglottic space. It is currently hypothesized that the tumor is derived from the internal branch of the superior laryngeal nerve [12].

The clinical picture of laryngeal schwannoma depends on the compressive effect of the mass on the larynx and surrounding organs. At first, the tumor can be asymptomatic, but as it grows, obstructive symptoms like hoarseness, dysphagia, and odynophagia start to appear [1]. Severe cases can present with life-threatening respiratory symptoms such as stridor and dyspnea with impending complete airway obstruction and can even lead to asphyxia [4].

Imaging is crucial in these tumors for accurate characterization of the mass. Laryngeal schwannoma usually is demonstrated as a round mass that is located medially to the thyroid cartilage and does not invade the surrounding tissues. The specific appearance of schwannoma on computed tomography (CT) is a submucosal, hypodense, localized mass that is well-defined [13]. On the other hand, magnetic resonance imaging (MRI) of schwannomas typically shows isointense to minimally hyperintense lesions in T1-weighted imaging and hyperintense lesions in both T2-weighted imaging and gadolinium-enhanced images [2].

Schwannomas can be definitively diagnosed by light microscopy under which specific histopathological characteristics can be visualized; Schwann cells typically aggregate as clusters with their nuclei being arranged in palisades, demonstrating the characteristic Antoni A pattern. Alternatively, they align together in edematous myxoid matrix with sparse cellularity (Antoni B pattern). When stained with S-100 stain, schwannomas exhibit a positive reaction [14].

Complete surgical resection is the main treatment strategy in schwannomas in order to prevent any potential recurrence. Other treatment options include CO_2_ laser microsurgery and endoscopic excision in patients with small tumors and a clear endolaryngeal view [1, 15]. Additionally, removal of schwannoma through transoral robotic surgery has been employed recently as a novel modality of treatment [16]. Tracheostomy and subsequently, lateral pharyngotomy, lateral laryngotomy, or laryngofissure approach are needed in patients with large tumors or a new recurrence [1].

## Conclusion

Laryngeal schwannoma is quite a rare, slow-growing tumor that is neurogenic in origin and presents with wide range of symptoms. Definitive diagnosis is achieved through visualization of characteristic pathological features of schwannoma under light microscopy. Treatment options include endoscopic and surgical excision of the tumor.

## Data Availability

Other data related to this case is available upon request from the corresponding author.
